# Atomic characterization of Si nanoclusters embedded in SiO_2 _by atom probe tomography

**DOI:** 10.1186/1556-276X-6-164

**Published:** 2011-02-23

**Authors:** Manuel Roussel, Etienne Talbot, Fabrice Gourbilleau, Philippe Pareige

**Affiliations:** 1 Groupe de Physique des Matériaux, Université et INSA de Rouen, UMR CNRS 6634, Av. de l'université, BP 12, 76801 Saint Etienne du Rouvray, France; 2 Centre de Recherche sur les Ions, les Matériaux et la Photonique (CIMAP), CEA/CNRS/ENSICAEN/UCBN, 6 Bd. Maréchal Juin, 14050 Caen Cedex 4, France

## Abstract

Silicon nanoclusters are of prime interest for new generation of optoelectronic and microelectronics components. Physical properties (light emission, carrier storage...) of systems using such nanoclusters are strongly dependent on nanostructural characteristics. These characteristics (size, composition, distribution, and interface nature) are until now obtained using conventional high-resolution analytic methods, such as high-resolution transmission electron microscopy, EFTEM, or EELS. In this article, a complementary technique, the atom probe tomography, was used for studying a multilayer (ML) system containing silicon clusters. Such a technique and its analysis give information on the structure at the atomic level and allow obtaining complementary information with respect to other techniques. A description of the different steps for such analysis: sample preparation, atom probe analysis, and data treatment are detailed. An atomic scale description of the Si nanoclusters/SiO_2 _ML will be fully described. This system is composed of 3.8-nm-thick SiO layers and 4-nm-thick SiO_2 _layers annealed 1 h at 900°C.

## Introduction

Since the discovery of photoluminescence of porous silicon by Canham in 1990 [1], nanostructured silicon systems have been extensively studied. Indeed, it exhibits properties (light emission, carrier storage, quantum confinement...) which lead to plenty of potential applications (photovoltaic cells, light amplifiers, nanoscale memory devices...) compatible with silicon integration technology [2-5]. Silicon nanoclusters (Si-nc) embedded in silica matrix is commonly considered as one of the most promising of these systems [6-9].

Si-ncs are usually produced by annealing silicon-rich silicon oxide (SRSO) to precipitate Si clusters in a silica matrix [10,11]. This SRSO can be obtained by different processes, such as ion implantation [12] or atomic deposition processes like chemical vapor deposition [13] and magnetron sputtering [14]. An efficient way to synthesize size-controlled Si-nc consists in sandwiching a SRSO layer between two SiO_2 _layers that prevent the excess of silicon from diffusing outside the SRSO film. Such a multilayer (ML) structure limits the Si-nc growth either during the growth or during the final step of annealing [15]. This fabrication process allows for controlling the major structural characteristics of the nanoclusters (size, composition, distribution and interface nature) for the achievement of the optimized optical properties of the device. Consequently, SRSO/SiO_2 _ML is a structure which has been intensively experimentally studied to quantify the correlation between Si-nc size and Si-nc properties [15-20]. However, conventional techniques suffer from drawbacks which prevent an accurate determination of the structure in these Si/SiO_2 _systems. Photoluminescence is one of the most usually used technique for such systems. Yet, it provides information only on the optical properties of Si-nc but no direct information about structural characteristics [21]. High-resolution transmission electron microscopy (HRTEM) for instance is not able to give satisfactory information about the composition of a particle and its surrounding chemistry and on the size distribution because misoriented and amorphous particles are excluded from the high-resolution image [21,22]. Most of the recent studies report the use of EFTEM to measure the size distribution of Si-nc [23-25]. As mentioned by Schamm et al., such size distribution measurements are based on the deconvolution of Si peak on EELS spectra. Besides, it gives only access to planar projection of three-dimensional (3D) objects, and Si-nc size strongly depends on data treatment and contrast enhancement. In addition, small clusters cannot be detected. These considerations lead to uncertainty as regards size distribution. Phase composition can also be extrapolated from EELS spectra. However, composition can only be determined under given assumptions like monodisperse Si-nc [24]. Finally, electron tomography has been performed by Yurtsever et al. [26]. This technique provides a 3D distribution of Si-nc. However, it does not allow quantitative composition measurements and can be tricky when it comes to small object (less than 1-2 nm). As the optical and electrical properties of nanocrystals are strongly dependent on these characteristics, a good understanding of phase separation and diffusion mechanisms will allow proposing a modeling of the growth and thus to improve the elaboration process at low cost. In order to achieve new information that complete or support the published one, atom probe tomography (APT) was performed in order to study the microstructure of SRSO/SiO_2 _MLs. This technique is able to provide a 3D chemical map of the sample at an atomic scale, allowing a very accurate and direct characterization of Si-nc in SiO_2_.

## Experimental

### SRSO/SiO_2 _MLs elaboration

SRSO/SiO_2 _MLs are synthesized by reactive magnetron sputtering. SiO_2 _pure targets are sputtered on 2" [100]-oriented wafer. Silica layers are deposited under pure argon plasma. As hydrogen has the ability to reduce oxygen, 50% H_2 _+ 50% Ar plasma is used to deposit SRSO layers containing approximately 50 at.% of silicon. The thickness of each layer is tuned by the sputtering time. After the deposition, HRTEM analysis allows for accurately calibrating the thicknesses of SiO_2 _and SRSO layers that are estimated to be 4 and 3.8 nm, respectively. The deposition process was fully described in a previous article [14]. Samples are deposited with a power density of 1.3 W cm^-2 ^at 650°C, and a first annealing treatment is realized after the deposition during 1 h at 900°C under N_2_. These conditions have already shown their efficiency to promote phase separation of the system.

### APT principle

APT is a powerful 3D chemical microscope which in principle relies on the field evaporation of surface atoms of a specimen and their identification by time-of-flight mass spectrometry. Since Müller et al. [27] invented the first atom probe in 1968, it has been used in materials science and particularly in physical metallurgy. Before the analysis, the specimen is prepared in the form of a sharp tip with a curvature radius less than 50 nm and placed under high vacuum (≈10^-13 ^Bar), at low temperature (80°K), at a high positive voltage (V_0 _≈ 5-15 kV). Under these conditions, an intense electric field is created at the apex of the tip (several V nm^-1^). Surface atoms are evaporated by means of electric pulses *V*_p _added to the DC voltage *V*_0 _and are collected on a position sensitive detector. The time of flight of each evaporated ion between the electric pulse and the impact on the detector is measured. This measurement permits calculating the mass-to-charge ratio:

(1) $$\frac{m}{n}=\frac{2eL^{2}}{t^{2}}\left(V_{0}+V_{\text{p}}\right)$$

where *m *is the mass of the evaporated ion (in kg), *n *its electronic charge, *L *the distance between the tip and the detector (in m), and *t *the time of flight of the ion (in s). This calculation permits identifying the chemical nature of evaporated ions. The use of some geometrical arguments and knowledge of the position of the impact of an ion on the detector permit calculating its position on the specimen, before the evaporation. These data enable the 3D reconstruction of the sample at the atomic scale. So far, the APT technique was restricted to metallic materials, but the recent implementation of femtosecond lasers permits the analysis of semi-conductors and dielectric materials. Instead of electric pulses, the ionization and the field evaporation of the surface atoms are triggered by the superposition of laser pulses. In this case, UV (343 nm) femtosecond laser pulses (50 nJ, 350 fs, 100 kHz) were used. In this study, APT analyses are carried out on a laser-assisted wide-angle tomographic atom probe (LA-WATAP) [28].

### Sample preparation for APT

As mentioned above, APT samples need to be prepared in the form of a sharp tip. The radius of curvature of the tip must be less than 50 nm to create a high electric field. The sample preparation is carried out using a focused ion beam (FIB) instrument. The Ga^+ ^ion beam is able to etch samples, and nanoscaled structures can be extracted from bulk materials. In order to prevent any Ga ions' implantation or sample degradation, a sacrificial platinum layer is deposited before every milling step (approximately 400 nm). This deposition is realized directly in the FIB instrument using the gas injection system.

**Figure 1:**
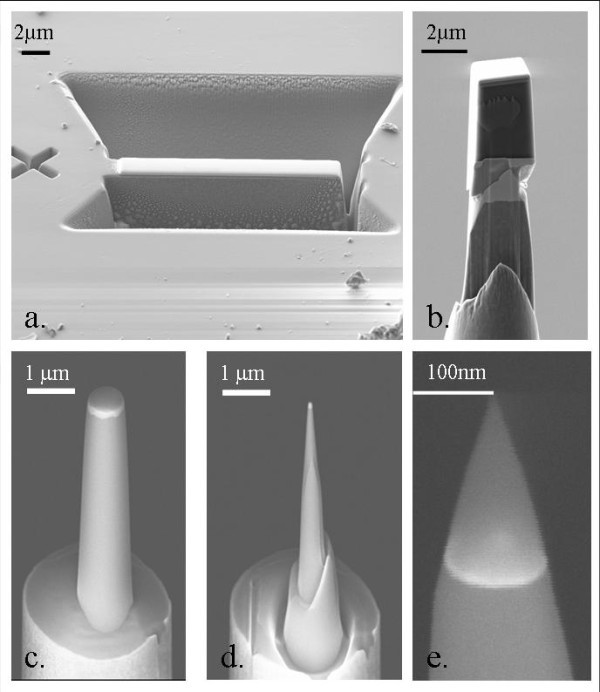
**FIB-SEM procedure for APT sample preparation**. **a**. Extraction of a silicon post using the Lift-out method. The sample has been milled with the help of a FIB in order to extract a strip of material. **b**. The strip is shaped in a post and welded onto a steel needle (platinum weld). **c**., **d**. and **e**. Successive annular milling steps permit to obtain a very sharp tip which curvature radius does not exceed 50nm.

The three-step method which is commonly used for obtaining a tip from the chunk state is illustrated Figure 1. The first step consists in etching a thin lamella of 2-4-μm-thickness in the sample (Figure 1a). Successive milling operations are operated on the chunk to extract posts [29]. The second step consists in micromanipulating and mounting extracted posts on the top of a stainless steel needle using a Pt weld (Figure 1b). During the final step, the post is submitted to an annular milling. The post is located along the axis of the ion beam which owing to annular motion successively cut concentric circles of the sample. By reducing the diameter of these circles, the post is thickened into a sharp tip with a curvature radius lower than 50 nm [30] (Figure 1c,d,e). To prevent ion beam damage and Ga implantation in the interest SRSO/SiO2 layers, the final milling is performed at a low accelerating voltage (2 kV). As observed in previous studies, this process ensures Ga-free tips [31,32].

## Results and discussions

**Figure 2:**
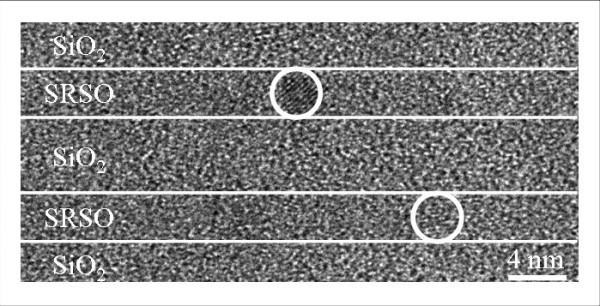
**HRTEM image of SiO_2_/SRSO layers in cross-sectional view**. White circles highlight two Si-ncs.

Before atom probe investigation, HRTEM images had been realized. An example is given in Figure 2. This image has been obtained on a Topcon 002B on samples prepared in a cross-sectional configuration. First, TEM analysis permits estimating the thickness of SRSO and SiO_2 _layers (3.8 and 4 nm, respectively). When diffraction conditions are obeyed, spherical crystalline clusters of silicon can be observed within the SRSO layers. Nevertheless, as noticed in former studies, only few Si-nc are evidenced by HRTEM. Therefore, it is difficult to determine an accurate size distribution, particle's density, and chemical composition of the matrix/precipitate interface. This kind of information can be obtained by APT.

### Evidencing Si-ncs

**Figure 3:**
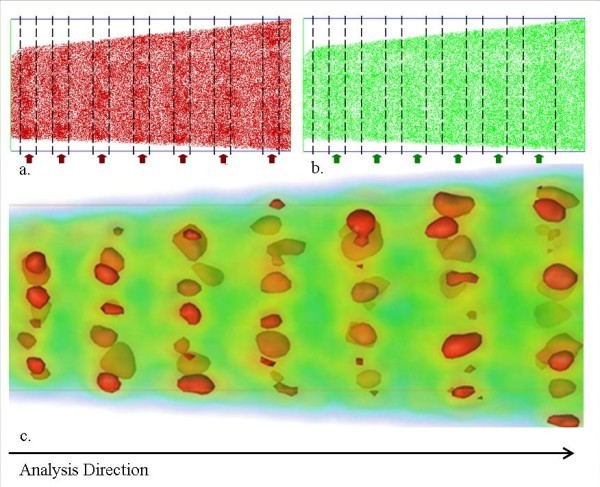
**3D reconstruction of SRSO/SiO2 MLs of APT analysis**. **a**. Distribution of silicon atoms in the analyzed volume. Each red dot corresponds to a silicon atom. Arrows indicate the location of SRSO layers. **b**. Oxygen atoms. Arrows indicate the location of SiO2 layers. **c**. Analyzed volume after cluster identification algorithm. Each red volume corresponds to silicon rich volumes (more than 75% of silicon) and green volumes correspond to silica composition (33% of silicon).

Figure 3 shows a 3D reconstruction of the same material analyzed by LA-WATAP. In Figure 3a,b, which represents the chemical map of Si and O atoms, each red dot corresponds to a silicon atom and each green dot corresponds to an oxygen one. The SRSO/SiO_2 _stacking sequence is clearly visible. In order to identify all the Si-nc (crystalline and amorphous), a cluster identification algorithm has been used. In this method, a sphere (1-nm radius) is placed over each atom of the volume, and the local composition is estimated by counting atoms within this sphere. The 3D reconstruction atoms, where the local concentration is above a given threshold (75 at.% in this case), permits revealing clearly Si-rich regions. A threshold of 33 at.% of Si can be used to evidence SiO_2 _regions. Figure 3c illustrates the result of this data treatment. Red volumes correspond to Si-nc and green ones to SiO_2 _matrix. Once this treatment is achieved, it is possible to estimate compositions of phases and interface, size distribution of Si-nc, and particle density in the analyzed volume.

### Composition information

**Figure 4:**
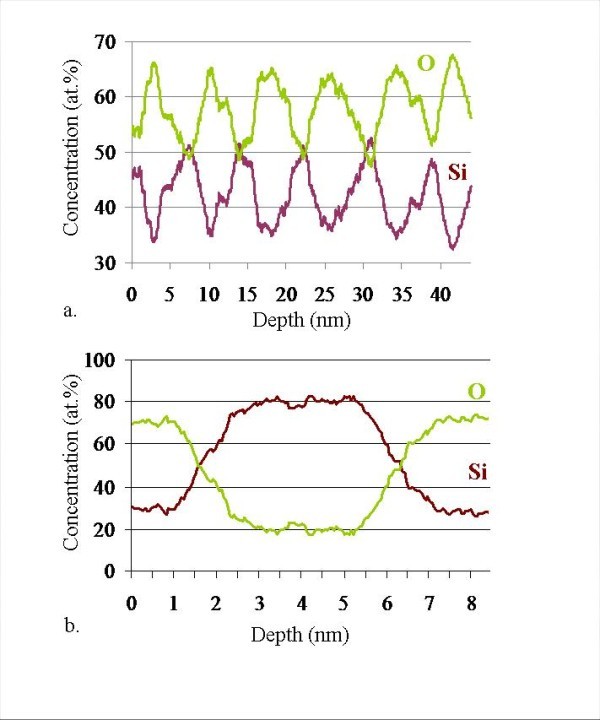
**Concentration profile deduced from APT experiments**. **a**. Concentration profile along the analyzed volume; **b**. Concentration profile across a precipitate.

APT analysis gives a chemical map of the sample and allows us to measure the composition of each phase. These compositions can easily be estimated by counting the atoms present into phases. The composition in SiO_2 _layers is estimated to be 34.3 ± 0.3 at.% of Si. This measurement is very close to the theoretical composition of SiO_2 _(33.3 at.% of Si). The composition of the SRSO layers is estimated to be 51.0 ± 0.3 at.% of Si which is very close to the composition of SRSO layers estimated during the elaboration process. This composition corresponds to a silicon excess of ≈26% in SiO_2_. The stacking sequence of silicon-rich and silica layers can be clearly identified on the composition profile realized along the axis of the analyzed volume (Figure 4a). APT technique gives a local composition at the atomic scale and allows us to study the phase separation within the SRSO layers. The annealing treatment (1 h at 900°C) realized on the samples induce the precipitation of the silicon excess. Two phases are observed: SiO_2_-matrix, and Si-precipitates. The matrix is composed of 41.9 ± 0.3 at.% of Si. This silicon concentration is significantly higher than in pure silica. An excess of silicon is still present in the matrix evidencing an incomplete phase separation between Si and SiO_2 _after the 1-h annealing at 900°C. Si-nc composition can be measured with the help of composition profiles as shown in Figure 4b. This composition profile shows the oxygen and silicon concentrations across a 4-nm-diameter Si-nc. Si-nc core compositions measured in this way systematically is 80 ± 3 at.% of Si for almost all clusters. This result is not coherent with HRTEM observations. Indeed previous electron microscopy studies have proven that Si-nc are pure silicon [21,22,33]. This difference is due to a well-known APT artifact: the local magnification effect. This effect is caused by the difference between the evaporation fields of Si-nc, which is significantly lower than that of the silica surrounding (matrix). It means that silicon atoms belonging to clusters evaporate more easily than silicon and oxygen atoms of the matrix, causing local variation of curvature radius and trajectory aberrations. Because of this phenomenon, some SiO_2 _is artificially introduced into Si-nc during the virtual reconstruction of the tip. The local magnification is well known in the APT community and can easily be corrected [34]. Talbot et al. [35] have proposed and used a correction to study matrix/cluster interface in SRSO layers.

### Size distribution and number density of Si-nc

**Figure 5:**
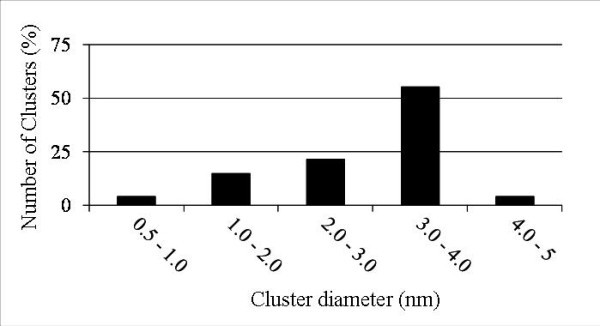
**Size distribution of Si-nc evidenced in the analyzed volume**.

In such nanostructured materials, one of the most important advantages of APT analyses is to be able to accurately measure the Si-nc size. Besides, since every precipitate is visible, it is possible to give a real size distribution which takes into account both crystalline and amorphous Si-nc. To estimate a precipitate's size, Si atoms within this precipitate are counted. From this number and assuming that Si-nc are spherical (as evidenced by HRTEM), it is possible to calculate the diameter for each cluster:

(2) $$d=2\times \sqrt[{3}]{\frac{3}{4\pi }.n_{\text{Si}}.\frac{V_{\text{Si}}}{Q}}$$

where *d *is the diameter of the particle (in m), *n*_Si _is the number of Si atoms in the particle, *V*_Si _is the atomic volume of a Si atom (in m^3^), and *Q *the efficiency of the detector (which is 50% in our case). The maximum error on such estimation is given by the variation of *d *associated to the number of Si atoms corrected from the local magnification effect. This error is about 0.1 nm for the smallest precipitate. Figure 5 shows a size distribution of clusters realized in the analyzed volume using this relation.

The size of the precipitates varies from 0.5 to 4.5 nm. The mean cluster diameter is 2.9 nm. More than 50% of the particle sizes lies in the range of 3-4 nm which is approximately the size of the SRSO sublayer (3.8 nm). The number density of particles is deduced from the number of particle in SRSO layers over these layers' volume. No cluster with a size greater than the SRSO layers was detected indicating that Si atoms in excess diffuse only in the SRSO layers. In this case, number density is estimated to be 9.0 × 10^18 ^± 1.0 × 10^18 ^cm^-3^. This density is very close to the theoretical number density of particles if all Si excess form precipitates of 3.8-nm-diameter with a layer thickness of 11.5 × 10^18 ^cm^-3^.

## Conclusions

In conclusion, APT has been used in this study to investigate SRSO/SiO_2 _ML containing Si-nc. We demonstrated that APT is able to provide a chemical map of such systems in 3D. Such analysis, at the atomic scale, allows for accurate and direct measurement of structural parameters like phase composition, size distribution, or chemical information on individual particle. For instance, it was established that for a 3.8-nm-thick SRSO containing 26% of silicon in excess, a 1 h of annealing treatment at 900°C induces the precipitation of Si-nc with a mean diameter of 2.9 nm and a number density of 9 × 10^18 ^cm^-3^. There remains 13% silicon excess in the SRSO layer, evidencing that phase separation is not complete. It can be assumed that further annealing treatment will result in the precipitation of the remaining Si excess, the increase of mean diameter, and the disappearance of small precipitates. Such information becomes easily accessible thanks to APT technique. Besides, such data are crucial to understand correlation between characteristics and photoluminescence or electrical properties of Si-nc, as well as the modeling of the kinetic of phase separation in these nanostructured systems, which are beneficial for the improvement of the elaboration processes.

## Competing interests

The authors declare that they have no competing interests.

## Authors' contributions

MR and ET carried out the APT sample preparation by SEM-FIB, performed and interpreted the APT experiments and wrote the manuscript. FG deposited the samples and performed HR-TEM experiments. PP supervised the study and participated in the analysis of the results. All authors read and approved the manuscript.
